# Modification of graphene oxide and its effect on properties of natural rubber/graphene oxide nanocomposites

**DOI:** 10.3762/bjnano.15.16

**Published:** 2024-02-05

**Authors:** Nghiem Thi Thuong, Le Dinh Quang, Vu Quoc Cuong, Cao Hong Ha, Nguyen Ba Lam, Seiichi Kawahara

**Affiliations:** 1 School of Chemical Engineering, Hanoi University of Science and Technology, No 1 Dai co Viet, Hanoi, Vietnam; 2 Faculty of Engineering, Nagaoka University of Technology, 1603-1, Kamitomioka-machi, Nagaoka, Niigata 940-2188, Japan

**Keywords:** graft copolymerization, graphene oxide, natural rubber, vinyltriethoxysilane

## Abstract

Modification of graphene oxide (GO) by vinyltriethoxysilane (VTES) was investigated to study the effect of silanized GO on radical graft copolymerization of GO onto deproteinized natural rubber (DPNR). The modified GO, GO-VTES (a and b), was characterized by X-ray diffraction (XRD), Fourier-transform infrared spectroscopy, contact angle, thermal gravimetric analysis, and scanning electron microscopy. The XRD results showed the appearance of an amorphous region of silica particles at a diffraction angle of 22°. The formation of silica was investigated by ^29^Si NMR, and it was found that the hydrolysis and condensation of VTES proceed more completely in basic conditions than in acidic conditions. The silica content of GO-VTES(b) was 43%, which is higher than that of GO-VTES(a) (8%). Morphology of silica was observed by SEM. The DPNR/GO-VTES nanocomposites prepared with the same amount of GO, GO-VTES(a), and GO-VTES(b) were characterized with tensile tests and dynamic mechanical tests. The stress at break of DPNR/GO-VTES(a) and DPNR/GO-VTES(b) was 5.2 MPa and 4.3 MPa, respectively, which were lower than that of DPNR/GO. However, it exhibited higher stress at small strains and higher storage modulus than DPNR/GO.

## Introduction

The graft copolymerization of natural rubber (NR) has gained significant interest for an extended period. This interest derives from the desire to enhance the green strength of NR to achieve the comparable mechanical properties of vulcanized natural rubber (VNR) [1–3]. VNR is commonly utilized for NR commercial products. However, due to its crosslinked structure, VNR products cannot be recycled or degraded after diposal [4–5]. Therefore, new approaches have been introduced to process NR into eco-friendly commercial products [6–7]. Since the discovery of the island nanomatrix structure of NR [8] and the development of effective methods to prepare deproteinized natural rubber (DPNR) [9–10], graft copolymerization of NR has had significant impacts on improving the mechanical properties of NR. Numerous studies have utilized suitable monomers to graft onto NR via radical routes. For instance, graft copolymerization of styrene [11–12], methyl methacrylate [13–14], and hydroxyethyl methacrylate [15–16] onto NR enhances the green strength for NR. On the other hand, the grafting of nanofiller onto NR to prepare NR nanocomposites has been recently discovered.

Nanofillers, such as nanosilica and derivates of nanocarbon, are the most well-known fillers that have been used in rubber technology. Nanosilica has been known to reduce the rolling resistance and enhance the wet grip of rubber materials. Recently, the usage of silane coupling agents as monomers to generate in situ nanosilica into NR has been found to enhance the mechanical properties of NR. Kawahara et al. [17] used vinyltriethoxysilane (VTES) as a silane monomer for grafting on NR to form an in situ nanosilica nanomatrix. The formation of nanosilica particles improved the thermal and mechanical properties of the graft copolymer. Furthermore, a well-controlled nanosilica nanomatrix structure in NR has been achieved in our previous work [18], where VTES was grafted onto NR grafted PS, gaining the best tensile strength at 19.23 MPa. This result highlights the advantage of a nanosilica nanomatrix on the improvement of mechanical properties of NR. On the other hand, graft copolymerization of nanocarbon materials, such as graphene and graphene oxide (GO) [19–20], has also attracted significant interest. This material with exceptionally high specific surface area, high mechanical properties, and high thermal conductivity is expected to prepare high-performance rubber composites [21–23]. In our recent work [24], we successfully designed a DPNR/GO composite by grafting GO onto DPNR. The results show that the tensile strength of DPNR/GO increased compared to that of DPNR, which proved the successful grafting of GO onto NR by the radical route. However, GO possesses a highly hydrophilic character, which is the opposite of the highly hydrophobic nature of NR. Thus, GO has quite poor compatibility with NR. It is necessary to modify GO to have a better interaction with the NR matrix.

Various researchers have attempted to functionalize GO with silane coupling agents to prepare hybrid GO/silica fillers [25–26]. The concept is based on the hydrolysis and condensation reaction of VTES to form nanosilica on GO membranes, using both acidic and basic conditions to catalyze these reactions. The hybrid GO/silica fillers could enhance the interaction, dispersion, and properties of various composites. For example, GO/3-aminopropyltriethoxysilane and GO/3-glycidyloxypropyltrimethoxysilane used in epoxy nanocomposites [27] or reduced GO/vinyltrimethoxy silane used in low-density polyethylene nanocomposites [28]. Additionally, Charoenchai et al. [29] prepared a hybrid silica/graphene oxide, GO/VTES, catalyzed under basic conditions. The hybrid filler was introduced into the NR matrix through conventional mixing on a two-roll mill. Mainly, GO/VTES was expected to improve its interaction with NR, thus enhancing the mechanical properties of NR at low deformation, along with other compressive and abrasive properties.

Until now, the application of hybrid silica/GO fillers has been limited to vulcanized NR. Moreover, nanoscale carbon-derived materials such as graphene and GO pose limitations in traditional NR vulcanization due to their associated high production costs. However, when ultilized in small quantities, nanoscale carbon-derived materials demonstrate potential in graft copolymerization of NR. Hence, the functionalization of GO with a silane coupling agent significantly contributes to exploring the most optimal methods and conditions for preparing NR/GO nanocomposites through graft copolymerization. Despite this, research involving the hybrid filler GO/silica for grafting onto NR remains unexplored. Therefore, applying this potential hybrid filler to enhance the green strength of NR through grafting techniques, forming a fine nanocomposite between NR and GO/silica hybrid filler, could be necessary. This approach may play a pivotal role in furthering the development of eco-friendly NR products.

In the present research, we modified GO with VTES and investigated the possibility of grafting GO-VTES onto NR through a radical route using tetraethylenepentamine (TEPA) and TBHPO as initiators. Exceptionally, GO-VTES was prepared by modifying GO with VTES under acidic and basic conditions to determine the ideal condition to modify GO for grafting onto NR. The GO-VTES products were characterized using X-ray diffraction (XRD), contact angle, ^29^Si NMR, Fourier-transform infrared spectroscopy (FTIR), and morphology analysis. The GO-VTES was expected to improve the mechanical properties of NR composite. The usage of GO-VTES may be suitable for the preparation of NR composites for tire applications as the composite may reduce water permeability and enhance the abrasion resistance of commercial products [30].

## Experimental

### Materials

The natural rubber used in this work is high-ammonia natural rubber latex (HANR, dry rubber content (DRC) 60%) supplied by Dau-Tieng rubber company. Sodium dodecyl sulfate (SDS, 97%) was bought from Nacalai Tesque, Japan. Graphite flake powder (>99% purity) was obtained from Yen-Bai province, Vietnam. The compounds KMnO_4_, NaNO_3_ (analytical grade), and TEPA were purchased from Sigma-Aldrich. Vinyltriethoxysilanes, tert-butyl hydroperoxide (TBHPO), and urea were purchased from Tokyo Chemical Industry, Japan.

### Removal of proteins from natural rubber

According to the method reported in [24], deproteinization of HANR was carried out using urea as a denaturing agent and SDS as a surfactant. About 80 g of HANR (60% DRC) was incubated with 80 g of water containing 1.6 g of SDS and 0.16 g of urea for 1 h before the 1st centrifugation at 10,000 rpm at 15 °C for 30 min. The cream recovered after centrifugation was redispersed in 80 g of water containing 0.8 g SDS and subjected to a 2nd centrifugation under the same condition. The cream fraction was again redispersed in water containing 0.16 g of SDS before the 3rd centrifugation. The final cream was diluted in a certain amount of SDS solution to obtain deproteinized natural rubber (DPNR) with DRC (20%) and SDS (0.2%).

### Preparation of graphene oxide and silanization with VTES

The graphene oxide used in this work was synthesized from graphite flakes using a modified version of Hummers’ method [31], similar to those reported in [24]. About 1 g of graphite powder was put into 23 mL of concentrated H_2_SO_4_, and 0.5 g of NaNO_3_ and 3 g of KMnO_4_ were added to the mixture. The temperature was maintained between 0–5 °C, and the system was kept for 2 h before heating up to 35 °C and kept for 1 h. After that, water was added to increase the temperature to 90 °C for 30 min, and 10 mL of H_2_O_2_ was dropped into the flask. After 15 min, 100 mL of water was used to dilute the reaction. The powder was filtered and washed with distilled water until it had a neutral pH. The GO powder was then dispersed in water and centrifuged at 10,000 rpm for 30 min to obtain the suspended solution. The purified GO powder was collected after drying the solution in a heating oven.

**Figure 1:**
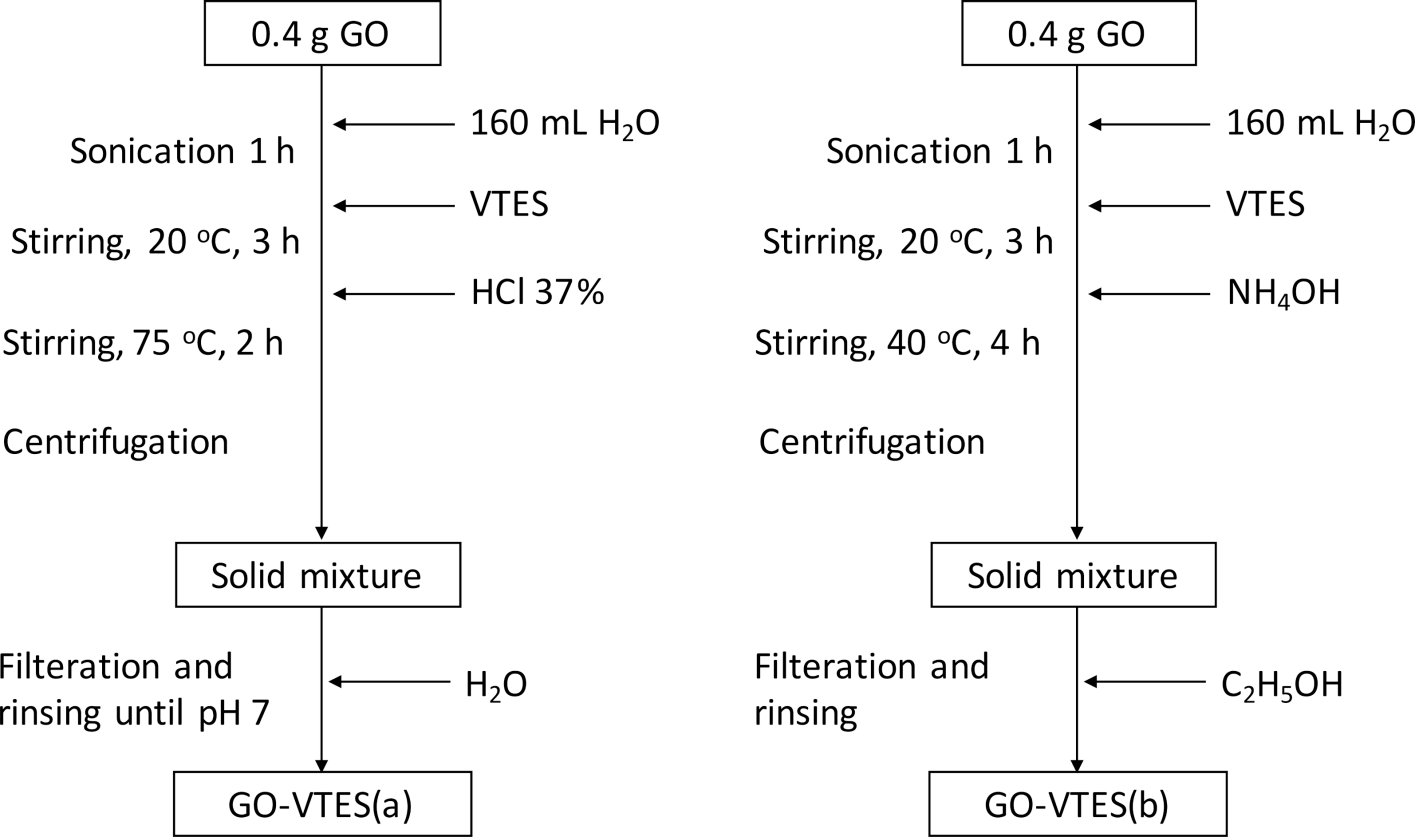
Silanization of graphene oxide in acidic condition (left) and in basic condition (right).

Vinyltriethoxysilane was used to modify GO [25,32] and it was bonded on the GO surface by the sol–gel method, as shown in Figure 1. Firstly, GO (0.4 g) was thoroughly dispersed in distilled water. After that, VTES (1.8 g) was added to the mixture, followed by the addition of concentrated HCl (1 mL) or NH_4_OH (1.5 mL) as catalysts. When using HCl, the mixture was stirred for 2 h under a temperature of 75 °C. Conversely, when NH_4_OH was used, the mixture was stirred at 40 °C for 4 h. The solid mixture, obtained after centrifugation, was rinsed and filtered until neutral. The collected solid was entirely dried in an oven at 50 °C to obtain the final GO-VTES(a) and GO-VTES(b).

### Preparation of DPNR/GO-VTES composite

**Figure 2:**
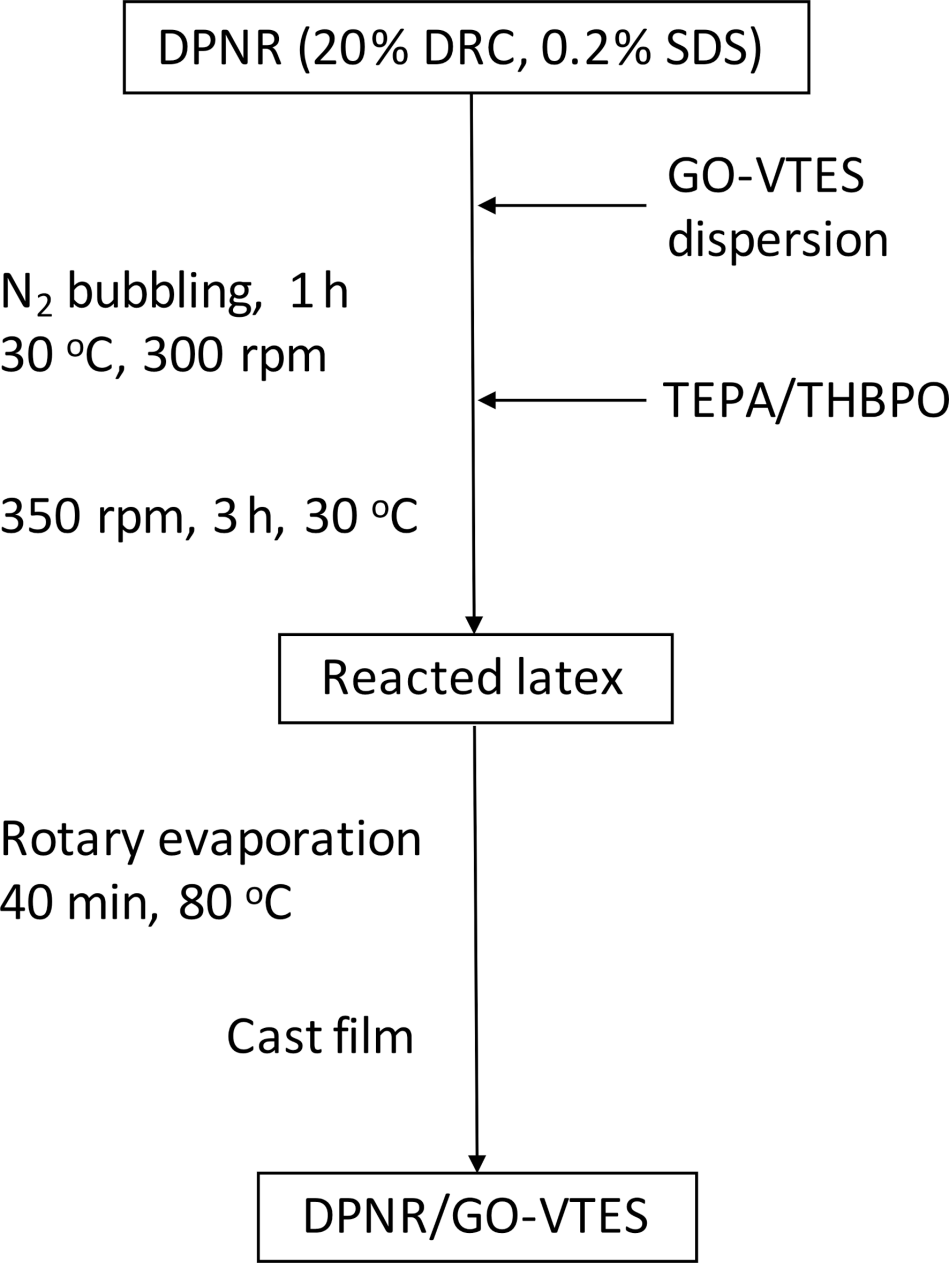
Preparation of DPNR/GO-VTES composite.

The DPNR/GO-VTES composite was prepared via graft copolymerization technique using TEPA/TBHPO redox initiators, similar to our previous work [24]. The experimental steps are illustrated in Figure 2. First, GO-VTES powder (0.2 g) was dispersed into 50 g of a 0.2% SDS solution followed by homogenization for 1 h to obtain a homogeneous GO-VTES dispersion. After that, approx. 200 g of DPNR (DRC 20%, SDS 0.2%) was mixed with the GO-VTES dispersion, and the system was bubbled with N_2_ gas for 1 h to remove residual dissolved oxygen. Then, 0.51 g of TEPA and 0.34 g of TBHPO initiators were added to the reaction to initiate the radical reaction between GO-VTES and the rubber particles. The reaction was carried out for 3 h with constant stirring under N_2_ atmosphere at 30 °C. The reacted latex was subjected to rotary evaporation for 40 min to remove impurities. The resulting latex was cast on a petri dish and dried in an oven at 50 °C, then in a vacuum to obtain a DPNR/GO-VTES composite film. A similar procedure was carried out with 0.2 g of GO, 200 g of DPNR (DRC 20%, SDS 0.2%), and with the same amount of TEPA/TBHPO as in the preparation of DPNR/GO-VTES.

### Characterizations

X-ray diffraction patterns of graphite and GO were acquired by using a Panalytical X'Pert Pro X-ray diffractometer. The measurements were performed at room temperature with the diffraction angle (2-theta) ranging from 5 to 80°.

Solid-state ^29^Si NMR spectra were recorded with a JNM ECA-400 (JEOL, Japan) spectrometer operating at a magnetic field of 400 MHz. A suitable amount of rubber sample was inserted in an NMR tube and injected into an NMR system equipped with a CP-MAS probe. The number of scans was 1000.

Contact angles of the samples were measured by taking a photo of a drop of distilled water on the sample surface by a CCD camera. The determination of the contact angle was monitored by the SCA20 software using the Data-physic OCA20 system.

Thermal gravimetric analysis (TGA) of the samples was performed on a TA Q500 instrument. The temperature was increased at a heating rate of 10 °C /min from room temperature to 900 °C in air atmosphere.

The morphology of the DPNR/GO-VTES was observed with field-emission scanning electron microscopy (FE-SEM) performed with HITACHI S-4800. The measurement was performed at room temperature.

The tensile properties of the composite samples were measured with a Tokyo Instron 5300 (Japan). The graft copolymer films (thickness of 1 mm) were cut in a dumbbell shape described in JIS K6251. Tensile measurements were done at room temperature with a speed test of 200 mm/min.

Dynamic mechanical analysis (DMA) of the graft copolymer was measured with a MCR 301 (Anton Paar Physica, Japan) analyzer using a 12 mm parallel plate (PP12) measuring system. First, the samples were heated to 80 °C in gaseous N_2_ and kept for 40 min for the attachment of the sample to the measuring system. The frequency-dependent measurement was performed at an initial strain of 1% and at an angular frequency of 0.1–100 rad/s at 30 °C. The number of data points collected was 31.

## Results and Discussion

### Characterization of GO-VTES

#### XRD

**Figure 3:**
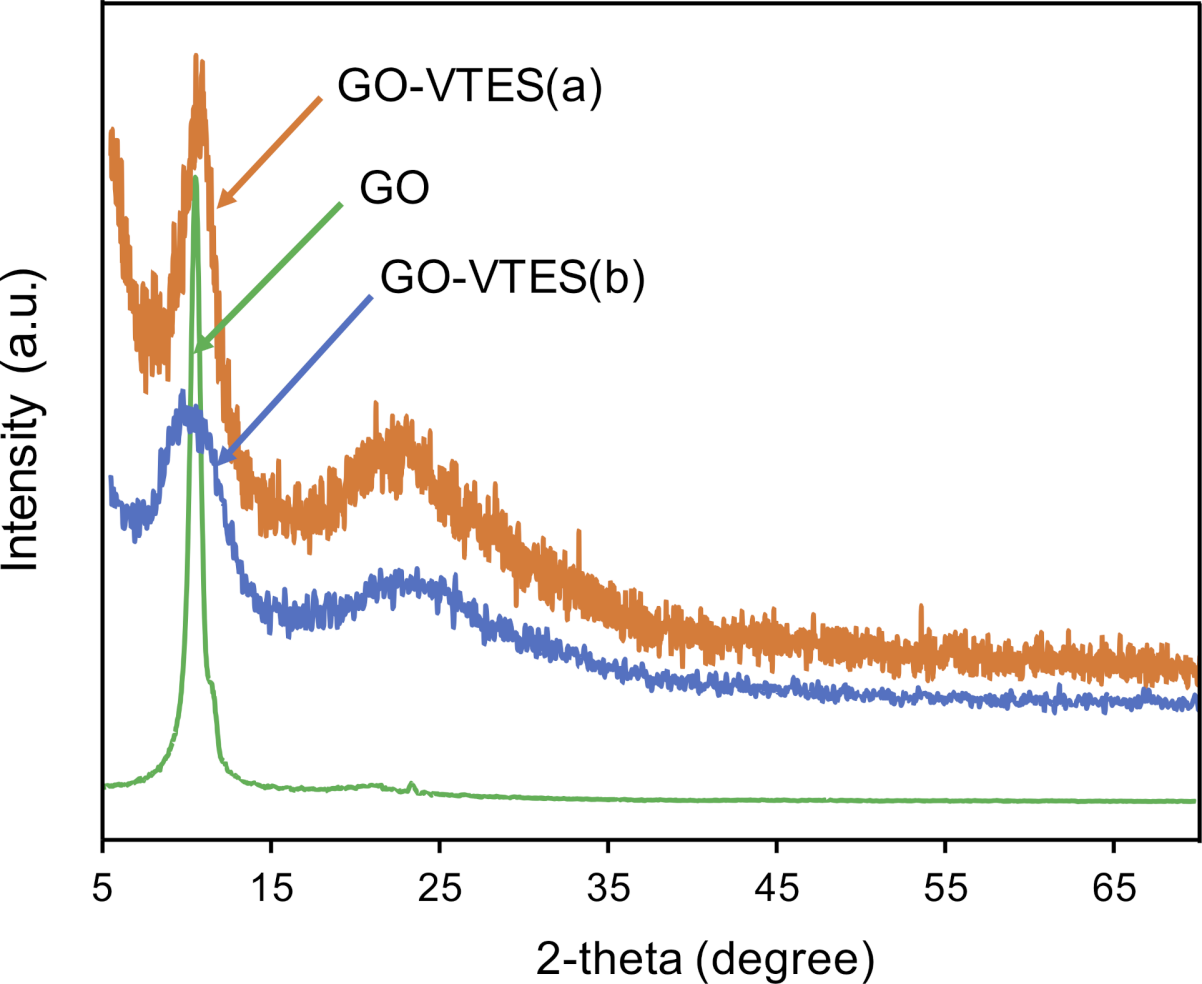
XRD patterns of GO, GO-VTES(a) and GO-VTES(b).

Figure 3 shows XRD patterns for GO and silanized GO using acid and base catalysts. The sharp peak at 2θ = 11° is a diffraction pattern for GO [33]. This peak is evidence of the exfoliation of the GO sheet synthesized by the modified Hummers’ method. After modification with VTES, two diffracting patterns appeared in the XRD spectra for GO-VTES(a) and GO-VTES(b). The peak at 2θ = 11° belongs to the GO sheet, and a new peak that appeared at 2θ = 22° was assigned to the amorphous silica. The broad peak of GO in GO-VTES may be explained to be due to the intercalation of the GO sheet during modification. Silica was formed after the hydrolysis and condensation of VTES. It is noted that the intensity ratio of the silica region and the GO peak for GO-VTEs(a) was lower than that of GO-VTES(b). This evidence may suggest that silica is more favorably formed in basic conditions than in acidic conditions. These results are a clear evidence for the formation of a silica layer on the surface of GO as a result of the reaction between GO and VTES.

#### ^29^Si NMR spectroscopy

**Figure 4:**
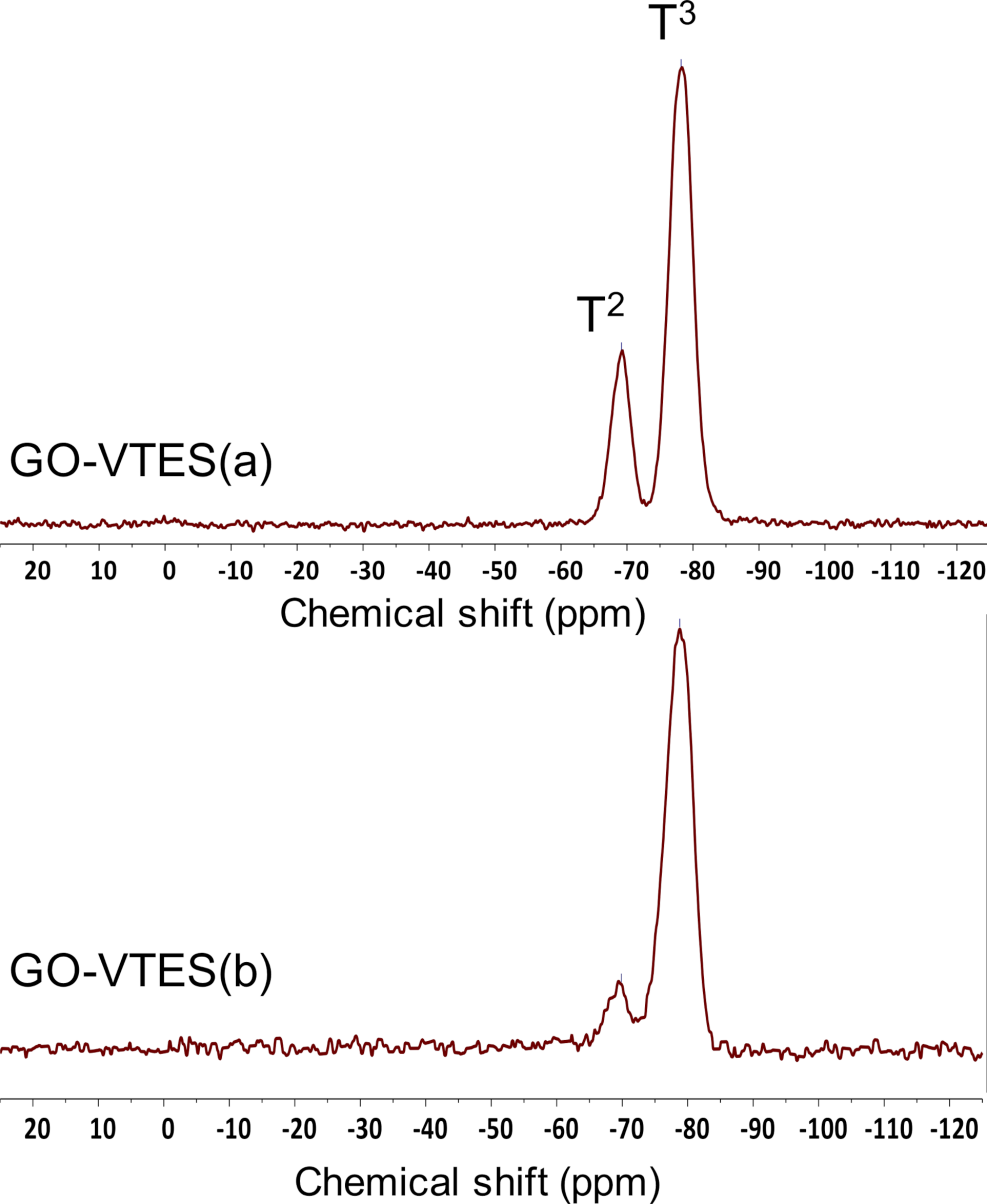
^29^Si NMR CP/MAS for GO-VTES(a) and GO-VTES(b).

To understand the degree of hydrolysis and condensation of VTES to form silica during GO modification, ^29^Si NMR spectra of GO-VTES(a) and GO-VTES(b) were recorded with a solid CP/MAS probe. In ^29^Si NMR spectra, the complete hydrolysis and condensation of VTES will result in the signal T_3_, corresponding to the R-Si(OSi)_3_ structure, and the incomplete hydrolysis and condensation of VTES will be accompanied by the T_2_ signal, which corresponds to R–Si(OSi)_2_OC_2_H_5_ or R–Si(OSi)_2_OH. As shown in Figure 4, two signals appeared at chemical shifts −69 and −79 ppm. The signal at −69 ppm was assigned to the Si atom in the T_2_ structure (i.e., CH_2_=CH–Si(O–Si)_2_(OC_2_H_5_) or CH_2_=CH–Si(O–Si)_2_(OH)). On the other hand, the signal at −79 ppm was assigned to the Si atom in the T_3_ structure (i.e., CH_2_=CH–Si(O–Si)_3_) [17]. It was noted that the intensity ratios of T_3_ to T_2_ signals for GO-VTES(b) were higher than that of GO-VTES(a). The higher intensity ratios of the T_3_ to T_2_ signals demonstrated that hydrolysis and condensation were more completed in GO-VTES(b) than in GO-VTES(a).

#### Attenuated total reflection–Fourier-transform infrared spectroscopy results

**Figure 5:**
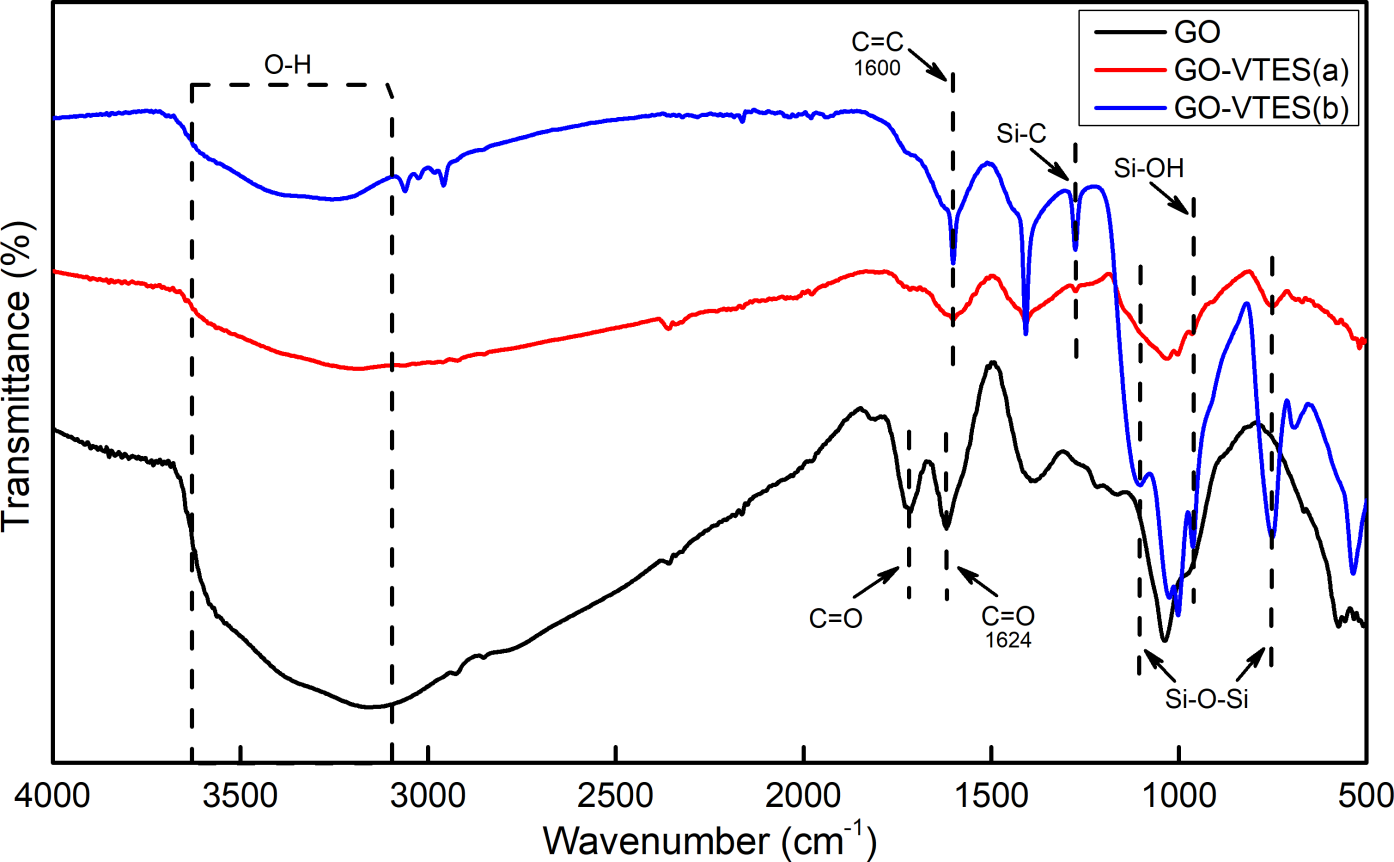
ATR–FTIR spectra of GO, GO-VTES(a) and GO-VTES(b).

The attenuated total reflection–Fourier-transform infrared spectroscopy (ATR–FTIR) results of GO, GO-VTES(a), and GO-VTES(b) are shown in Figure 5. The absorption values ranging from 3100 to 3700 cm^−1^ corresponded to –OH and –COOH groups on the GO surfaces and –OH group on silica particle surfaces [33]. However, the intensities of these signals decreased in the FTIR spectra of GO-VTES(a) and GO-VTES(b). This may be attributed to a decrease in the number of –OH groups of GO due to the reaction with VTES and the presence of the vinyl group on the surface of silica particles. The peak at the wavenumber of 1720 cm^−1^ in the GO spectrum corresponds to the C=O bond in the carboxylic group –COOH. However, after modification with VTES, the intensity of the 1720 cm^−1^ signal significantly decreased. It may be due to the removal of the –COOH group during GO modification. In the spectrum of GO, the absorption peak at 1624 cm^−1^ was ascribed to vibration of the C=O bonds on the ketone group [33]. Nevertheless, for GO-VTES(a) and GO-VTES(b), this absorption peak overlapped with the absorption peaks at 1600 cm^−1^, which appeared due to the appearance of C=C bonds in VTES [17]. In the fingerprint region, the absorption peaks at approx. 950 to 1300 cm^−1^ for GO were due to the vibration of C–O and C–OH bonds in GO. These absorption peaks decreased for GO-VTES(a) and increased for GO-VTES(b) after modification. The increased in these peaks for GO-VTES(b) was due to the asymmetrical vibration of Si–O–Si linkages in silica particles during modification. This suggested that the silica particles were more efficiently formed in basic conditions than in acidic conditions. Note that the absorption at 780 cm^−1^ was due to the symmetrical vibration of Si–O and Si–C linkages. This absorption was due to the formation of silica particles on the GO layer during modification.

#### Contact angle

**Figure 6:**
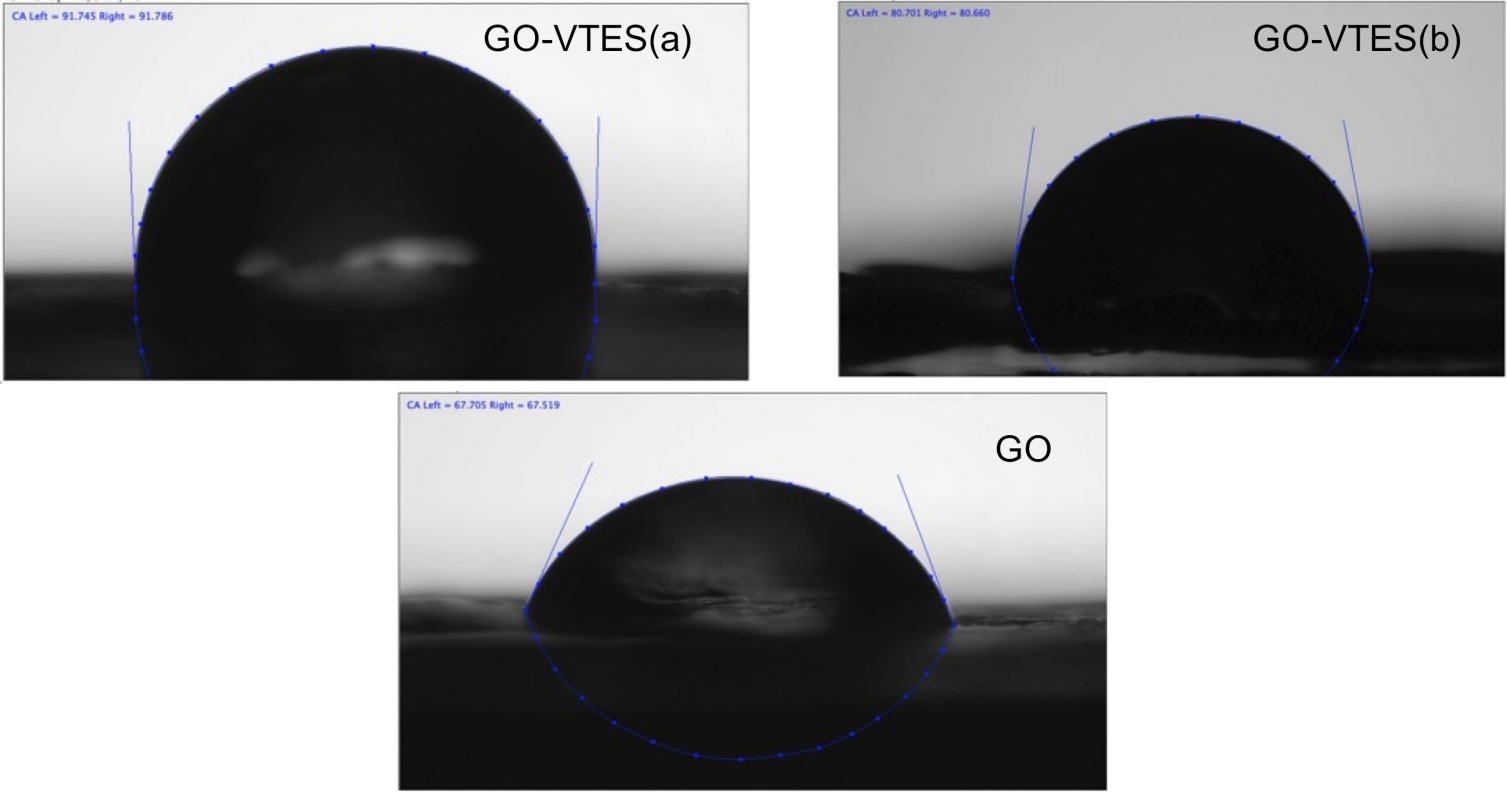
Contact angles of GO, GO-VTES(a) and GO-VTES(b).

The effect of GO modification with VTES on GO surface characteristics was investigated by measuring the contact angle. Figure 6 shows images of distilled water that was dropped onto GO and GO-VTES surfaces. The value of the contact angle elucidates the hydrophilicity or hydrophobicity of the surface. The higher the contact angle, the higher the hydrophobicity. The contact angle of GO is about 67°, and those of GO-VTES(a) and GO-VTES(b) are 91 and 80°, respectively. The excellent hydrophilicity of GO may be due to various hydrophilic functional groups (i.e., hydroxyl group, carboxyl group, and epoxy group) that exist on the GO surface. However, after modification with VTES, the contact angle increased. This suggests that the formation of silica particles enhanced the hydrophobicity of GO. Note that GO-VTES(a) has a higher contact angle than that of GO-VTES(b), which indicates that GO-VTES(a) exhibited higher hydrophobicity than GO-VTES(b). It may be due to a vinyl group (–CH=CH_2_) or ethoxy group (–OC_2_H_5_) attached to the silica surface. However, in the case of GO-VTES(a), the hydrolysis was not completed. Thus, there were fewer –OH groups on the silica and GO surfaces. This result is in agreement with the ^29^Si NMR and ATR FTIR analysis.

#### Thermal gravimetric analysis results

**Figure 7:**
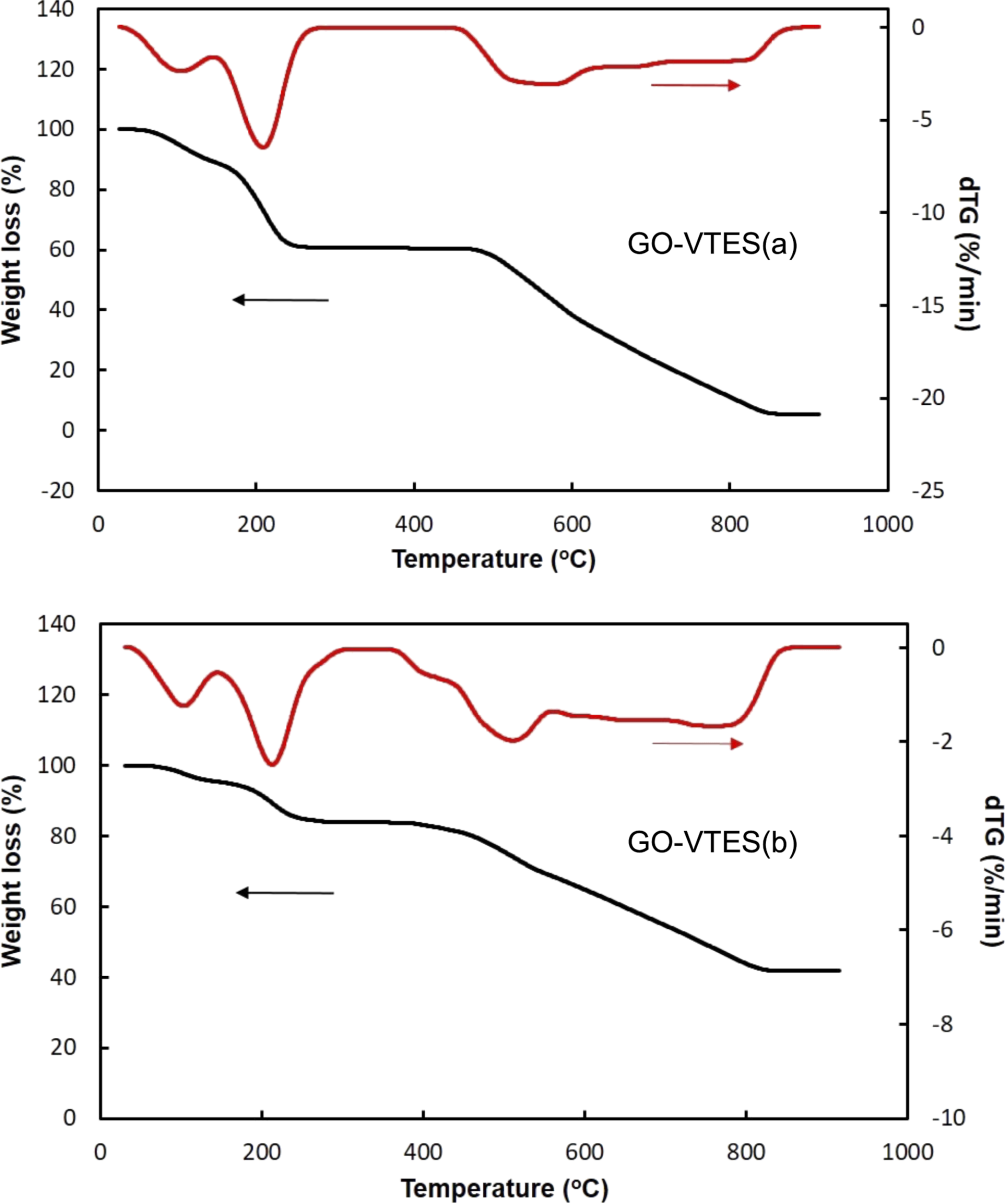
TGA curve of GO-VTES(a) and GO-VTES(b).

The degradation behavior of GO-VTES was investigated with TGA. Figure 7 shows TGA and dTGA curves for GO-VTES(a), and GO-VTES(b) and the weight loss percentage at three temperature ranges and residual ash are given in Table 1. The weight loss percentage from 30 to 100 °C for GO-VTES(a) and GO-VTES(b) was approx. 6% and 4%, respectively. This weight loss was attributed to absorbed water in GO-VTES. At higher temperatures ranging from 150 to 250 °C, the weight loss was due to the decomposition of oxygenous organic functional groups present in GO-VTES. The weight loss percentage in this temperature range was approx. 34% for GO-VTES(a) and 12% for GO-VTES(b). The higher weight loss percentage for GO-VTES(a) may be due to the higher amount of combustible functional groups derived from incomplete hydrolysis of VTES. These results confirmed that silica formation is more favorable in a basic medium than in an acidic medium. From 500 to 800 °C, the combustion of carbon-containing compounds occurs. The weight loss of GO-VTES(a) and GO-VTES(b) were quite similar, approx. 52% and 42%, respectively. After 800 °C, the residual ash was silica. The ash content was 8% for GO-VTES(a) and 42% for GO-VTES(b). From the ash content, it could be confirmed that the VTES was efficiently attached to GO, and the attachment was more efficient in basic conditions rather than acidic conditions.

**Table 1:** Weight loss percentage of GO-VTES(a) and GO-VTES(b) at different temperatures.

Sample	Weight loss (%)			

	30 to 100 °C	150 to 250 °C	500 to 800 °C	Residual amount

GO-VTES(a)	6	34	52	8
GO-VTES(b)	4	12	42	42

#### Scanning electron microscopy images

**Figure 8:**
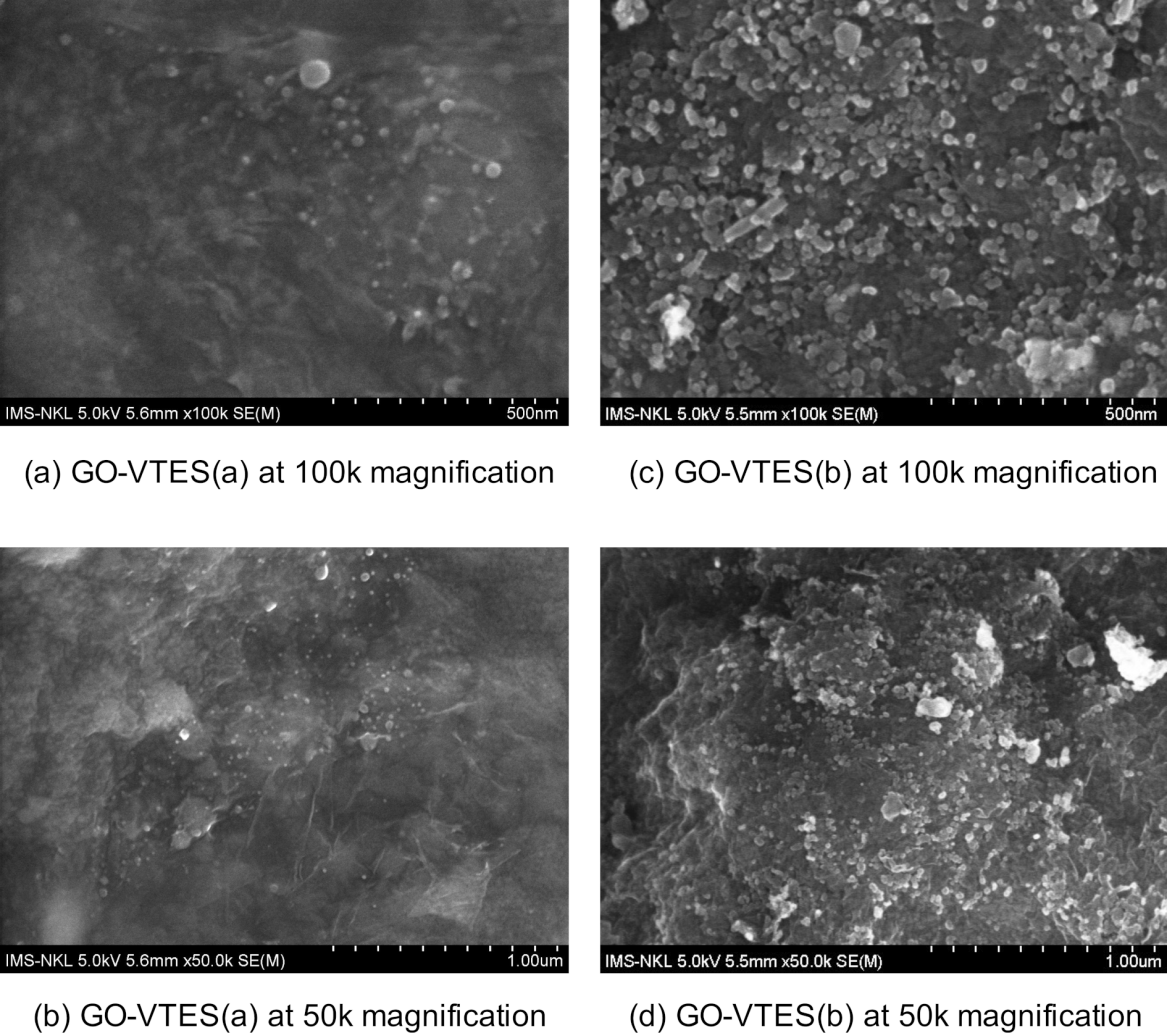
FE-SEM images for GO-VTES(a) and GO-VTES(b) at 100k and 50k magnifications.

Figure 8 shows SEM images for GO-VTES(a) and GO-VTES(b). As can be seen, silica was produced with a size of approx. 50 nm for GO-VTES(a) and GO-VTES(b). It proved that VTES was hydrolyzed and condensed to generate the nanosilica particles. Moreover, more silica particles were found in SEM images of GO-VTES(b) than in GO-VTES(a). At the same time, GO sheets were more visible in the case of GO-VTES(a) but entirely invisible in GO-VTES(b). Nanosilica was generated and covered most of the GO surface in GO-VTES(b), and there was some small agglomerates of silica.

#### Illustration of GO modification with VTES

**Figure 9:**
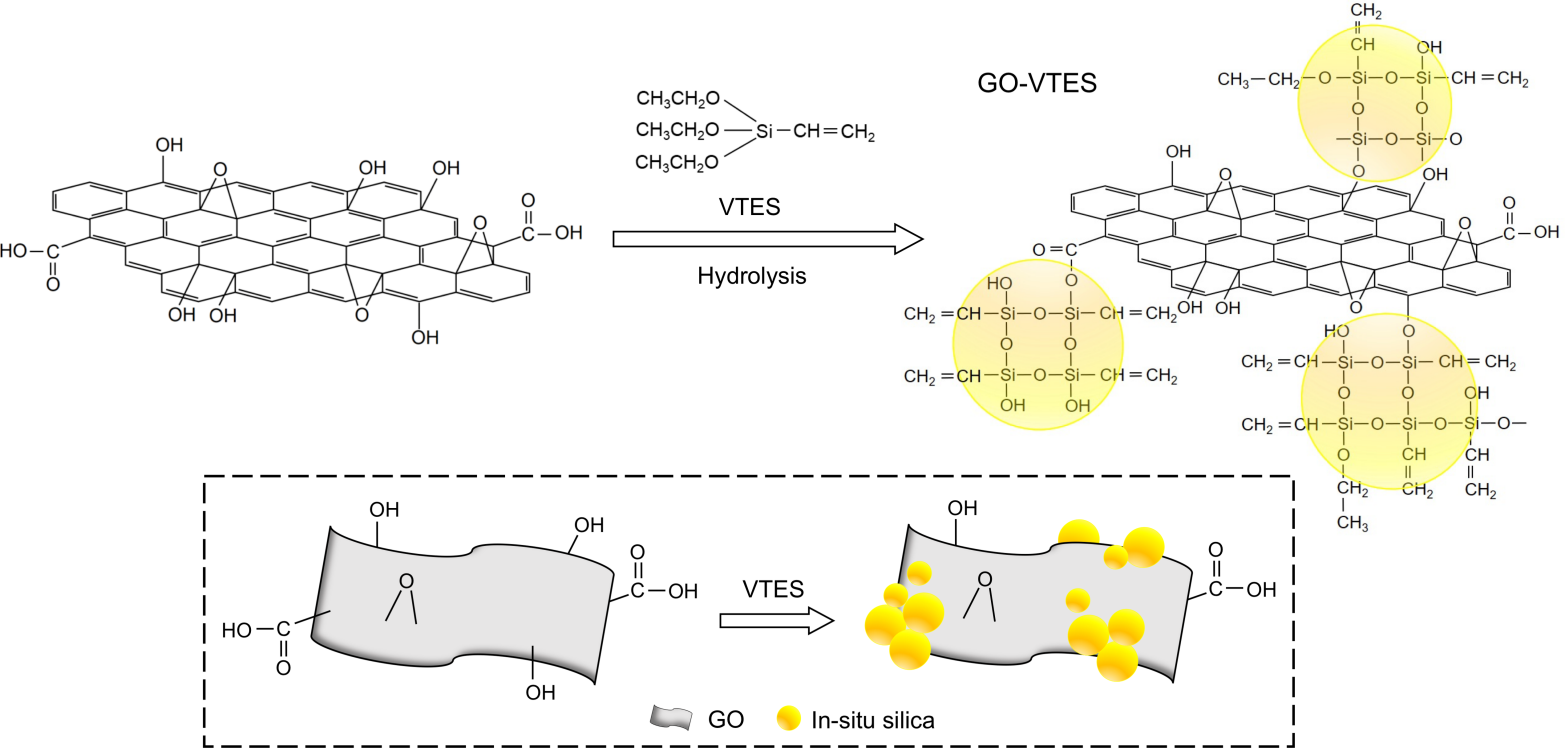
Schematic for modification of GO-VTES.

Figure 9 illustrates GO modification with VTES. First, VTES is hydrolyzed to form –OH groups. These –OH groups will be attached to the GO surface at the hydroxyl group by hydrogen or chemical bonding. The formation of silica particles from hydrolysis and condensation of VTES may occur on the GO surface and in water. The unreacted vinyl group in GO-VTES may have possible interactions with rubber particles through radical graft copolymerization, same as those reported on graft copolymerization of VTES onto NR [17–18].

### Properties of DPNR/GO-VTES

#### Stress–strain curves

**Figure 10:**
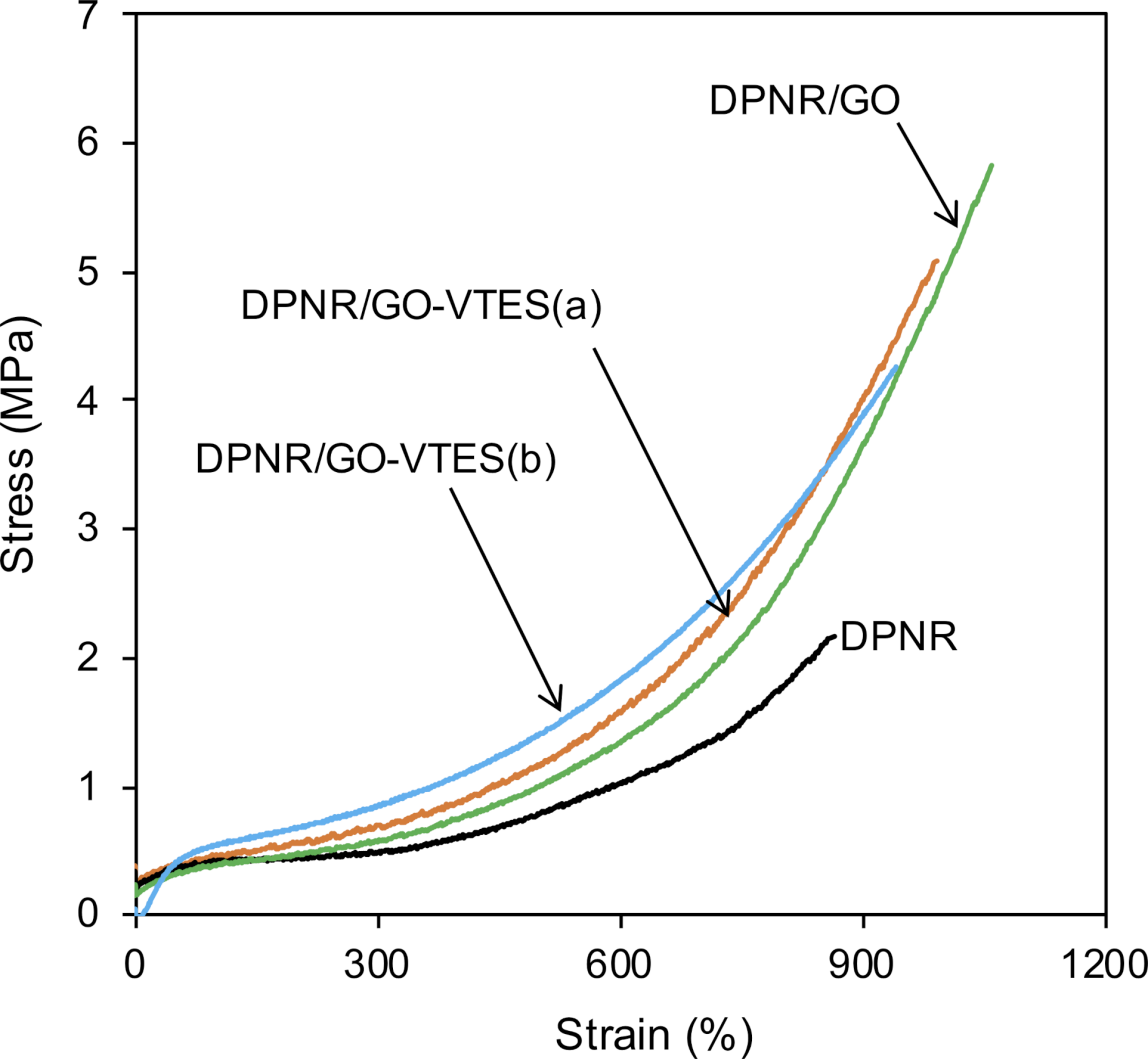
Stress–strain curves of DPNR, DPNR/GO, DPNR/GO-VTES(a), and DPNR/GO-VTEs(b).

Figure 10 shows stress–strain curves for DPNR/GO-VTES(a), GO-VTES(b), and DPNR/GO as a control sample. The amount of GO, GO-VTES(a), and GO-VTES(b) used in graft copolymerization was 0.5 phr. The tensile strength of the unmodified DPNR was approx. 2.2 MPa. It increased to 5.2 MPa for DPNR/GO-VTES(a) and to 4.3 MPa for DPNR/GO-VTES(b). However, these tensile strength values were slightly lower than that of DPNR/GO, which was 5.9 MPa. This observation may indicate that GO, after modification with VTES, was not well chemically linked to rubber particles, resulting in a decrease in the reinforcement effect for NR. The giant silica structure on the GO surface, as proposed in Figure 9, may hinder the mobility of GO to interact with rubber particles during radical graft copolymerization. Table 2 shows the value of stress at strain of 100%, 300%, 500%, stress at break, and strain at break for DPNR/GO-VTES(a), DPNR/GO-VTES(b), and DPNR/GO. The samples DPNR/GO-VTES(a) and DPNR/GO-VTES(b) have higher stress values at 100%, 300%, and 500% than those of DPNR/GO. This was due to silica particles on the GO surface, providing the stiffness for DPNR/GO-VTES. Furthermore, tensile strength for DPNR/GO-VTES(a) was slightly higher than that of DPNR/GO-VTES(b), which suggested that GO-VTES(a) may have better interaction with NR than GO-VTES(b). The silica particles on the GO layer may hinder the chemical interaction between GO and NR.

**Table 2:** Tensile stress at break, strain at break, tensile stress at strain of 100%, 300%, 500% of DPNR, DPNR/GO and DPNR/GO-VTES samples.

Sample	Tensile stress at a strain of (MPa)			Tensile stress at break (MPa)	Strain at break (%)

	100%	300%	500%		

DPNR	0.39	0.47	0.77	2.2	864
DPNR/GO	0.39	0.59	1.00	5.8	1060
DPNR/GO-VTES(a)	0.47	0.69	1.16	5.1	1006
DPNR/GO-VTES(b)	0.55	0.86	1.40	4.2	941

#### Dynamic mechanical properties

**Figure 11:**
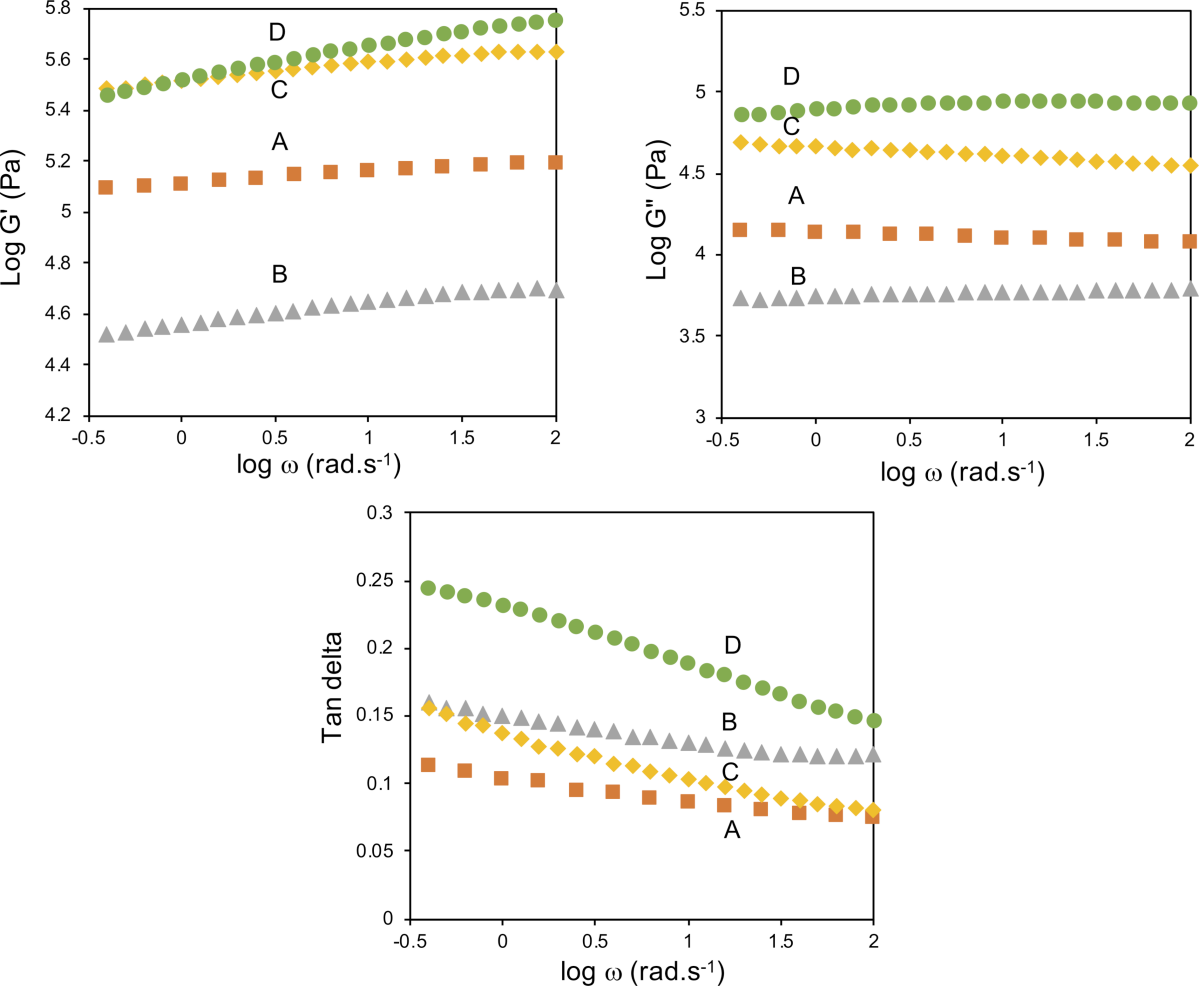
Dependence of storage modulus (G'), loss modulus (G''), and loss tangent (tan δ) of (A) DPNR, (B) DPNR/GO, (C) DPNR/GO-VTES(a), and (D) DPNR/GO-VTES(b) as a function of frequency.

Dynamic mechanical properties of composite samples reveal how much energy is stored or lost during applied cyclic shearing force. Figure 11 shows the dependence of the storage modulus (G'), loss modulus (G''), and loss tangent (tan δ) of DPNR, DPNR/GO, DPNR/GO-VTES(a), and DPNR/GO-VTES(b) as a function of frequency at 30 °C. The G' value of DPNR was 0.13 MPa. This value was much higher than that of DPNR/GO0.5 (0.036 MPa). However, it was lower than those of DPNR/GO-VTES(a) (0.284 MPa) and that of DPNR/GO-VTES(b) (0.334 MPa). The lower G' value of DPNR/GO could be explained by thin and large surface GO layers. The GO sheet could not withstand large shearing force, causing the rubber particles to slip. The high G' value of DPNR/GO-VTES(a) and DPNR/GO-VTES(b) may be due to hard silica particles, which may contribute to higher energy storage for composite materials. The G' values seemed to depend on the silica content; the higher the silica content, the higher the storage modulus.

The dependence of loss modulus (G'') on frequency for DPNR, DPNR/GO, DPNR/GO-VTES(a), and DPNR/GO-VTES(b) exhibited a little difference as shown in Figure 11. The G'' values of DPNR and DPNR/GO-VTES(a) slightly decreased with frequency. However, the G'' values for the DPNR/GO and DPNR/GO-VTES(b) samples slightly increased as frequency increased. These distinguishable behaviors suggested that DPNR/GO and DPNR/GO-VTES(b) may have higher energy dissipation while applying force than DPNR/GO-VTES(a) and DPNR.

The loss tangent (tan δ) is defined as a G''/G' ratio. The dependence of tan δ with frequency for DPNR/GO samples was quite similar to that of DPNR, in which tan δ decreased as frequency decreased. This phenomenon is appointed to the pure entropic elasticity of NR. Notably, values of tan δ for DPNR/GO-VTES(a) and DPNR/GO-VTES(b) sharply decreased with frequency. This suggests that the energy may be dissipated faster in DPNR/GO-VTES(a) and DPNR/GO-VTES(b) than in DPNR and DPNR/GO. The absence of chemical interactions between NR and GO-VTES in DPNR/GO-VTES(a) and DPNR/GO-VTES(b) may be responsible for a fast energy dissipation. Consequently, DPNR/GO0.5 which uses unmodified GO, shows both entropic and energetic elastic properties, as evidenced by the dependence of loss tangent versus frequency [34].

## Conclusion

Graphene oxide was modified by VTES under acidic and basic conditions to produce GO-VTES(a) and GO-VTES(b), respectively. The GO-VTES(a) and GO-VTES(b) samples were then used for the preparation of nanocomposites with deproteinized natural rubber via the graft copolymerization route. The DPNR/GO-VTES(a) and DPNR/GO-VTES(b) samples exhibited lower tensile strength values than that of DPNR/GO. However, they showed higher stress values at small strain values than those of DPNR/GO. This demonstrates that GO-VTES may have poorer chemical interaction with DPNR than GO. From DMA analysis, DPNR/GO-VTES(a) and DPNR/GO-VTES(b) may have more viscous characteristics than DPNR/GO. The silica particles in GO may decrease the chemical interaction between GO and NR via graft copolymerization. However, it contributed to a higher storage modulus for DPNR/GO-VTES(a) and DPNR/GO-VTES(b), which were approx. two or three times higher than that of DPNR/GO. Our work provided insight into how modified GO interacts with rubber particles during radical graft copolymerization compared to unmodified GO. Then, a suitable approach could be made in the future to fabricate NR/silica/GO composite for abrasion resistance.
